# Inulin as a Fat-Reduction Ingredient in Pork and Chicken Meatballs: Its Effects on Physicochemical Characteristics and Consumer Perceptions

**DOI:** 10.3390/foods11081066

**Published:** 2022-04-07

**Authors:** Leidy Montoya, Natalia Quintero, Stevens Ortiz, Juan Lopera, Patricia Millán, Aída Rodríguez-Stouvenel

**Affiliations:** School of Food Engineering, Universidad del Valle, Cali 76001, Colombia; leidy.montoya@correounivalle.edu.co (L.M.); quintero.natalia@correounivalle.edu.co (N.Q.); brayan.ortiz@correounivalle.edu.co (S.O.); juan.lopera@correounivalle.edu.co (J.L.); patricia.millan@correounivalle.edu.co (P.M.)

**Keywords:** chicken, consumer test, FOS, inulin, meatball, penalty analysis, pork, product development, reduced-fat

## Abstract

Fat reduction in meat products represents a technological challenge, as it affects the physicochemical and sensory properties of foods. The objective of the present investigation was to develop reduced-fat pork and chicken meatballs. In the initial stage, a survey was performed on 387 individuals, in order to determine the consumer perception of the meaning of a healthy meatball and the likelihood that they would consume such a product. In the second stage, four pork and chicken meatball formulations were developed: control meatballs (AC), meatballs with inulin (AI), meatballs with fructo-oligosaccharides (AF), and meatballs with inulin and fructo-oligosaccharides (AM). In the third stage, physicochemical properties were evaluated (water activity, humidity, fat, protein, ash, weight loss, pH, color, and texture) and a sensorial profile was created with semi-trained panelists for the four meatball formulations. In the fourth stage, AI was selected as the meatball with sensorial and physicochemical characteristics most similar to AC. An analysis of nutritional characteristics and a home test (84 consumers) were performed. The present study established that the inclusion of inulin as a fat substitute in the preparation of pork and chicken meatballs, in the amount of 3.5 g of fiber/100 g of the mixture, imitates the technological properties characteristic of fat and showed acceptance by consumers.

## 1. Introduction

International nutrition policies are encouraging the reformulation of meat products either by eliminating or reducing some ingredients or incorporating bioactive compounds. The World Health Organization (WHO) recommends that fats should not exceed 30% of calories in the diet. Of this percentage, saturated fats should only encompass 10% of calories, and cholesterol consumption should be limited to 300 mg/day. In addition, the elimination of trans-fatty acid from the food supply are suggested by WHO [1]. In accordance with Resolution 810 of 2021 of the Colombian Ministry for Social Protection [2], which establishes nutritional and food labelling regulations, the Colombian food industry will begin to implement warning statements for foods high in saturated fat, sodium, and added sugar.

Researchers and processed meat producers seek healthier products by way of reducing nutritional deficiencies, without affecting product sensorial qualities or healthiness [3]. The options to the reformulation of meat products include the addition of antioxidants [4,5,6,7], sodium reduction [8,9], addition of dietary fiber [10,11,12,13], incorporation of monounsaturated and polyunsaturated fats [14,15,16,17] and fat reduction [18,19,20,21,22,23,24].

Fat replacement in meat products is achieved by the use of lean meat and the addition of water, in higher levels than in traditional products, as well as the addition of other ingredients with little or no caloric content [25]. Research in the development of reduced-fat meat products includes the use of cereal and legume fibers, β-glucans, starches, gums, pectin, aloe vera, and prebiotics. The development of processed meat faces a technological challenge, in terms of fat reduction, as this significantly alters the sensorial characteristics thereof, as fat serves important functions in the determination of three principal sensorial characteristics: appearance (color and surface uniformity), texture (viscosity, elasticity, and firmness), and flavor intensity. [13,25,26,27,28].

Fiber has been employed recently for the development of reduced-fat meat products, given the textural and organoleptic characteristics that it contributes, as well as the reduced caloric value and nutritional effects [29,30,31]. The fat substitution by canola and olive oils has caused greater hardness in low-fat hamburger meat; however, the incorporation of inulin (3.1%) and β-glucan (2.2%) could reduce this undesirable effect on the sensorial properties of hamburgers [14]. In addition, adding prebiotics to meat may improve the technological and sensorial characteristics of hamburgers [26]. The oatmeal β-glucans (OG) and their hydrolysates (OGH) as fat substitutes has been used in the production of pork meatballs, obtaining improved overall acceptance between samples [18]. Rye and pea fiber affect the sensorial qualities of meatballs resulting in gritty texture, less juiciness and crumbly texture for pea fiber meatballs [32].

Even though palatability in meat products has changed over time, tenderness, juiciness, and flavor are still considered important aspects for acceptability and preference for cooked meat. This is not only determined by the meat, in and of itself, but also by demographic aspects [33]. The development of healthier meat products represents a technological challenge that are finally evaluated by consumers, as when they are purchased, characteristics such as aroma, appearance, texture, and flavor become decisive factors. It is important to identify the way in which consumers perceive the sensorial attributes of food products, as demonstrated by their growing participation in the characterization of foods. [34,35]. The objective of the present study was to develop reduced-fat pork and chicken meatballs and to apply sensorial analysis methodologies to measure consumer perceptions of this type of product.

## 2. Materials and Methods

### 2.1. Consumer Sensorial Perceptions during Meatball Consumption

In order to evaluate the stimuli with descriptions of meatballs of different types, the Polizer et al. [36] methodology was employed. This was carried out with traditional meatballs (Stimulus A), one that was reduced in fat (Stimulus B), one with fiber (Stimulus C), and one without preservatives (Stimulus D). By way of a virtual format, the stimuli were presented to participants following the monadic presentation, and they were instructed to evaluate their perceptions, as related to both health and probability of consumption. One structured scale was utilized for each of the two evaluations, in accordance with Table 1.

Data were collected from 387 individuals. They were recruited in Colombia, by way of social networks, email, and telephone. These individuals were chosen in accordance with the following characteristics: they were all over 18 years of age, consumers of pork and chicken, consumed processed meat products at least biweekly, and were from social division by strata on three, four, and five. Participants consumed meat products at least once per month, 84.5% of whom were in the age range of 18–45 years, 61.5% were women, 38.5% were men, 78% had undergraduate and/or postgraduate degrees, and 72.61% resided in the Valle del Cauca, Colombia.

### 2.2. Raw Materials for Meatball Preparation

Meats were purchased at a local supermarket. The chicken breast, lean pork loin, and bacon used complied with the quality requirements, in accordance with National Colombian Standards and Regulations [37,38,39,40,41]. High-purity refined salt (Refisal), parboiled P.A.N. white corn flour (Polar Inc.), paprika (Stravaganza), powdered oregano (El cocinerito), powdered bay leaf (El Rey), powdered garlic, pure ground thyme, powdered basil, powdered ginger, ground pepper, inulin, oligofructose, and Carl natural color (Tecnas- Ingredient Company at Colombia) were used.

### 2.3. Meatball Preparation

Meatballs are defined as processed meat products, whether cooked or raw, that are not packed as sausage, and are prepared with meat, the addition of substances whose use is permitted [42]. They were prepared in the Experimental Kitchen area at the Unitary Food Engineering Operations at the Universidad del Valle (Colombia). Previous studies with focal groups determined the base formulation for meatball preparation as well as concentrations of added fiber. The meats were received and stored at −18 °C, until their use. The remaining ingredients were stored in a fresh, dry area, at room temperature. Raw materials were weighed, in accordance with this formulation, for the creation of 320 meatballs in 1 kg batches (48 meatballs). The chicken breast, lean pork loin, and bacon fat were ground, using a meat grinder (ESSEN), and were added to a mixer (Kitchen Aid), consecutively adding the spices, salt, color, water, and corn flour over the course of five minutes. The meat mixture was stored in the freezer until it reached 4 ± 1 °C, in order to permit uniform shapes. Pieces of the meat mixture were weighed in amounts of 20.5 ± 0.5, using an Adventurer (OHASUS) scale. Meatballs were formed manually into spheres, and were boiled in water at 85 ± 2 °C, until their internal temperature rose to 80 ± 1 °C. They were then extracted from the water, drained for 30 s, and subjected to a thermal shock until their internal temperatures dropped to 35 ± 1 °C. Meatballs were packed on PET/WINPACK easy-open, transparent, thermoformed plastic trays measuring 615 µm and a PET/Coextruded. HBA (PE-EVOH) sheet measuring 80 µm, using a modified atmosphere of 40% CO_2_ and 60% N_2_.

Table 2 shows the formulations which correspond AC control meatball samples (neither reduced in fat, nor with added fiber), AI inulin meatballs (with reduced fat and added inulin dissolved in water), AF meatballs with fructo-oligosaccharides (with reduced fat and added FOS dissolved in water), and AM mixed meatballs (with reduced fat and added inulin and FOS dissolved in water). In the AI, AF, and AM samples, fat was replaced with the addition of 3.5 g of fiber (inulin and/or FOS) dissolved in water in a 1:2 proportion (fiber: water).

### 2.4. Measurement of Physicochemical and Microbiological Parameters

Water activity. Water activity measurement occurred in accordance with the Association of Official Agricultural Chemists AOAC 978.19 method [43], using an AquaLab Vapor Sorption Analyzer Hygrometer VSA (METER Group, Washington, DC, USA). Slices of meatball centers were obtained and placed in the sample cup. Measurements were taken in triplicate.

Meatball pH. This measurement was taken in accordance with the AOAC 981.12 method [44], in which 10 g of meatballs were weighed, placed in a blender, 90 mL of distilled water was added, and these were blended for one minute. This was filtered, and a meat suspension was obtained, from which the pH measurement was taken, using an Orion Star A215 pHmeter (Thermo Scientific, Madrid, Spain). Measurements were taken in triplicate.

Humidity content. The preparation of the meat sample was performed in accordance with those methods established in the AOAC 983.18 [45], beginning with 200 g of ground meatball sample. The humidity determination occurred via an air stove drying method according to National Colombian Standard NTC 1663 [46], using a Binder stove, and placing 5 ± 0.01 g of sample at 125 ± 1 °C for five hours. Measurements were taken in triplicate.

Color. Chromatic properties were evaluated in septuplicate, in the crust and internal areas of each meatball, in accordance with those criteria described in International Organization for Standardization ISO 7724-2 [47] using a CM-5 Chroma Meter spectrophotometer (Konica Minolta Business Technologies, Tokyo, Japan), and as a reference, illuminant D 65 and 10° observer.

Texture measurement. The texture measurement occurred using texture analysis equipment (EZ-SX, Shimadzu Corp., Kyoto, Japan) with a load cell of 500 N and a five-blade Kramer cell. Measurements were taken in septuplicate, and samples were placed at 25 °C. Four meatballs were placed into the cell, the blade displacement velocity was 50 mm/min.

Physicochemical analysis. Total protein—ISO 1871:2009 [48], total fat—ether extract AOAC 920.39C [49], ash—AOAC 920.153 [50], fiber—AOAC 999.03 [51], fatty acid profile AOAC 969.33 [52], cholesterol—AOAC 976.26 [53], iron and sodium—AOAC 984.27/ICP—OES [54]. All measurements were taken in triplicate.

Microbiological analysis. Recount of aerobic mesophiles—AOAC 966.23 [55], NMP of total coliforms—ICMSF:2000 [56], recount of *Staphylococcus* positive coagulase—UNE EN ISO 6888 [57], *Salmonella* detection in 25 g—UNE EN ISO 6579 [58], recount of *Clostridium* spores reducing sulphite—ICMSF:2000 [56], detection of *Listeria monocytogenes*—UNE EN ISO 11290 [59] and detection of *Escherichia coli*—ISO 4832 [60] were all performed in triplicate.

### 2.5. Meatball Sensorial Profile

The descriptive analysis of the meatballs was performed in 60 sessions of 1–3 h, in accordance with the methodology established by ISO [61], NTC [62,63,64,65], and Kemp et al. [66]. A total of 8 candidates were selected based on: (i) normal acuity for the different senses include non-existence of physical or physiological deficiencies; (ii) discriminative ability, evaluated through of triangular and ranking test; and (iii) ability to describe their sensory perceptions. In order to establish the evaluation scale, a consensus was reached between panelists for descriptors. The intensities of the descriptors were determined using a non-structured 10 cm long scale next to each descriptor that was anchored on the left side, for the terms “slight”, “none”, and “light”, and on the right side, “strong”, “a lot”, “dark”, and “high” (Table 3). The methodology indicated by Saldaña et al. [67] and Quadros et al. [68] was used to define both the scale and references.

The objective of the training consisted of the memorization of references at the extremes of each attribute. The panel’s performance and final evaluator selection occurred considering three criteria: discrimination, reproducibility, and consensus. The final evaluation occurred in accordance with the vocabulary developed by the panel, using three meatballs per session. The panel consisted of eight judges, who evaluated 13 sensorial attributes related to appearance, aroma, texture, and flavor.

### 2.6. Consumer Sensorial Evaluation of Meatballs

**Figure 1:**
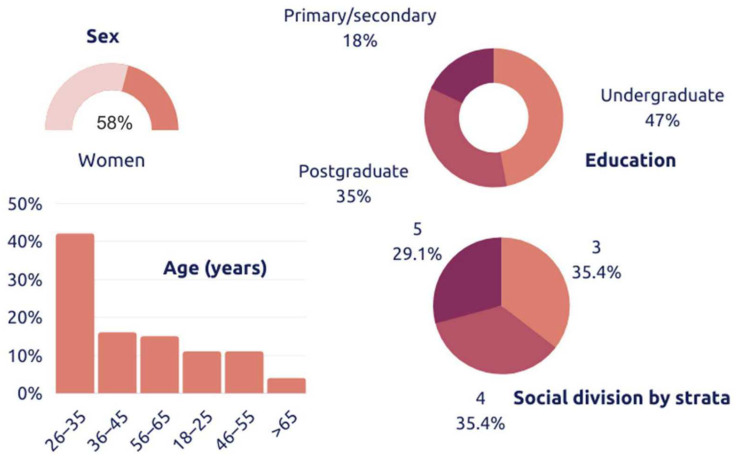
Descriptive statistics of consumers who resided in the Valle del Cauca, Colombia. (*n* = 84).

The sensorial evaluation of reduced-fat meatballs occurred by way of a home test. A total of 84 consumers were recruited at Cali (City of Colombia), by way of social networks, email, and telephone. These individuals were chosen in accordance with the following characteristics: they were all over 18 years of age, consumers of pork and chicken, consumed processed meat products at least biweekly, were not allergic to any of the product ingredients, and were from strata three, four, and five. The descriptive statistics of consumers are shown in the Figure 1. Samples were distributed the day after their preparation, and meatballs were guaranteed to be kept in portable refrigerators during delivery to each home. One tray of meatballs, packed in modified atmospheres, and one 100 g packet of ranchera sauce were provided. Preference studies performed previously, with 100 consumers, had established that this type of sauce was considered adequate as an accompaniment to pork and chicken meatballs. Each individual signed informed consent, and received a product evaluation survey with the instructions to be followed. On the form, it indicated that the meatball was already cooked and ready for consumption, and that it only required heating. In the tasting process, the meatballs could be heated together with a moderate amount of ranchera sauce, or could be prepared in the way in which the individual wished to consume these (with pasta, Bolognese sauce, without sauce, etc.).

The scale structure was designed considering NTC 5328 [65]. All panelists evaluated the samples in terms of overall acceptance, appearance, aroma, texture, and meatball flavor, on a nine-point hedonic scale (9 = I like them very much, 1 = I strongly dislike them). In addition, a three-point “JAR Just About Right” scale was used to evaluate color, firmness, juiciness, saltiness, the intensity of the spiced flavor, and fat level (3 = more than I like, 2 = just as I like, 1 = less than I like), and an open question was prepared, such that panelists could describe the reasons for which they did or did not like the samples evaluated. At the end, the consumer was asked to indicate how healthy they considered the meatballs to be (on a scale of 1–7, where 1 was not at all healthy, and 7 was very healthy) and the probability that they would purchase them (on a scale of 1–7, where 1 was not at all probable, and 7 was very probable). The Penalty Analysis was performed in accordance with the methodology indicated by Concha-Meyer et al. [69], Davis et al. [70], Naes et al. [71], and Lawless et al. [72].

### 2.7. Statistical Analysis

A completely randomized, unifactorial design, with fixed effects, and two replicates was employed. The factor which corresponded to fiber and the four levels which corresponded to the different types of meatball formulations with fiber were as follows: inulin, FOS, mixture (inulin-FOS), and control (fiberless meatball). All results were subjected to Kolmogorov–Smirnov and Shapiro–Wilk normality tests occurred by way of SPSS statistical software (IBM SPSS v25.0, Chicago, IL, USA).

The physico-chemical results were analyzed using ANOVA and the Tukey test (parametric statistics). For the data of probability of consumption and perception of healthiness for meatballs a paired comparison test (nonparametric statistics) was used. The data during the training of the panelists were analyzed by means of binomial test, adjusted chi-square, normal distribution test and Walds analysis (parametric statistics). Sensory profile data of the 4 meatball formulations evaluated by trained panelists were analyzed by ANOVA. The hedonic data from the consumers were analyzed using nonparametric statistics (Kruskall–Wallis test) because they did not have a normal distribution. The penalty analysis for the sensorial analysis occurred by way of Xlstat software (Addinsoft, Paris, France), with which “Just About Right” scale (JAR) results permitted the identification of those attributes which influenced overall acceptance scores for meatballs with inulin. Variables were considered significant with *p* < 0.05, and differences were determined based on the Tukey HSD test.

## 3. Results

### 3.1. Consumer Sensorial Comparison of Meatball Consumption

**Figure 2:**
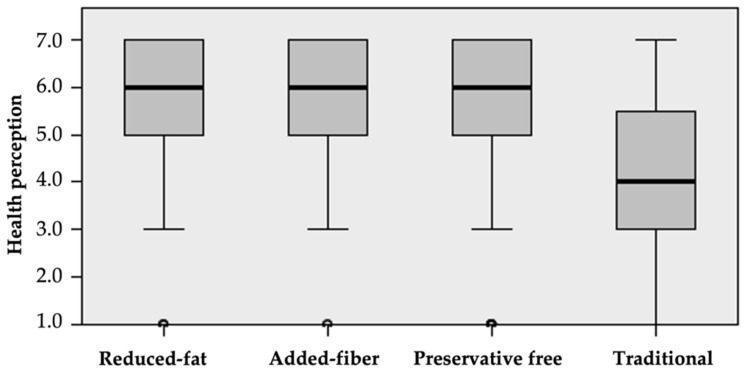
Median test for health perception of meatballs.

Consumers considered the reduced-fat, preservative free, and added-fiber meatballs to be equally healthy. However, they perceived these meatballs to be significantly healthier (median = 6.0) than traditional meatballs (median = 4.0) (Figure 2 and Table 4).

**Figure 3:**
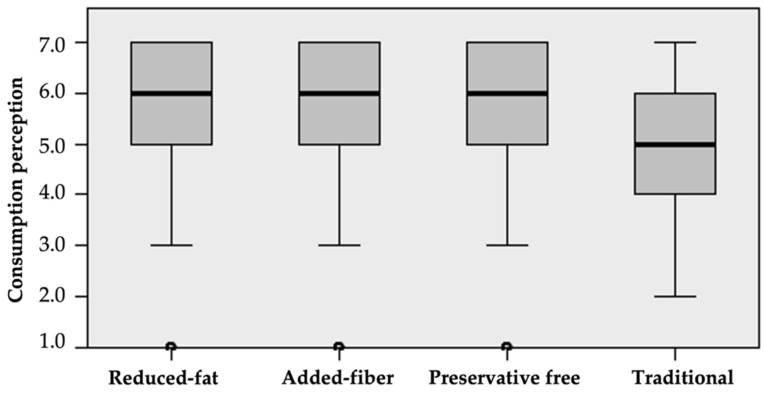
Median test for probability of meatball consumption.

Figure 3 identifies that there is a significantly higher probability of meatball consumption when they have added fiber, are reduced in fat, or are preservative free (median = 6.0), than a traditional meatball (median = 5.0). There is a greater tendency to consume preservative-free meatballs than those with added fiber, whereas the probability of consumption is equal between added-fiber and reduced-fat meatballs, as well as for reduced-fat and preservative-free meatballs (Table 5).

In the “Voice of the Industry: Food & Nutrition” survey, performed by Euromonitor in 2021, it was found that the functionality of foods as key components in well-being has become an important element for consumers. Over 75% of industry professionals hope that individuals focus much more on health and well-being in the next five years, given the impact of COVID-19 on the way in which consumers eat [73]. The results obtained in the present study indicate that new global consumer tendencies are also reflected on a national level, where reduced-fat meatballs are considered healthier than traditional meatballs, and as such, these are more likely to be consumed.

The preservative free term is perhaps the most important for consumers. The preservative free meatballs were predominantly perceived as healthy relative to other associations, and as such, their probability of consumption is also greater. In accordance with the information reported by Mintel, the main meat product launches in 2020 were food apt for microwaves (28.1%), that were additive/preservative free (20%), low in/without allergens (18.2%), easy to use/prepare (17.6%), and gluten free (16.8%) [74].

### 3.2. Physicochemical, Microbiological Characteristics and Sensorial Profiles (Trained Panelists) of Four Meatball Formulations (AC, AI, AF, AM)

Table 6 shows the results of the physicochemical characteristics of pork and chicken meatballs. Protein and ash contents did not present significant differences between samples, while differences in fat content were directly related to the amount of added fat in the AI, AF, and AM formulations. Color parameters (L, a, b, C, H) in the internal and external parts of the meatballs did not present significant differences between treatments, except for luminosity of the internal part of samples AI, AF, and AM, which were higher than in AC.

The fat reduction and addition of fiber-water significantly affected (*p* < 0.05) meatball humidity content, and neither affected the water activity thereof, nor the pH. The loss of weight was lower in AC than in AI or AF, and was highest in AM. Kramer maximum force was significantly higher for AC meatballs than added-fiber meatballs. This could be due to the fact that fiber prevented the rigidity characteristic of reduced-fat meat products.

Certain soluble fibers are used as fat imitators, as is the case of inulin, a prebiotic dietary fiber with broad use as a fat replacement in various food matrices, it improves viscosity, forms gels, increases water-retention capacity, and improves mouth texture [30]. In the present study, the addition of water, together with fiber, may explain many of significant differences in physicochemical parameters and between treatments. It caused less toughness, greater humidity, and loss of weight, compared to the AC control sample (Table 6).

Observe that, in Table 7, the pork and chicken meatballs comply with the microbiological requirements of Technical Colombian Regulation 1325 [42], in terms of the recount of mesophilic aerobes (CFU), Coliforms (CFU), *Staphylococcus aureus* (CFU), *Salmonella* sp./25 g of sample, *C. perfringens reducing sulphite* (CFU/g), *Listeria monocytogenes*/25 g of sample, and *E. coli*, indicating that it is an innocuous product, and as such, is apt for consumption.

**Figure 4:**
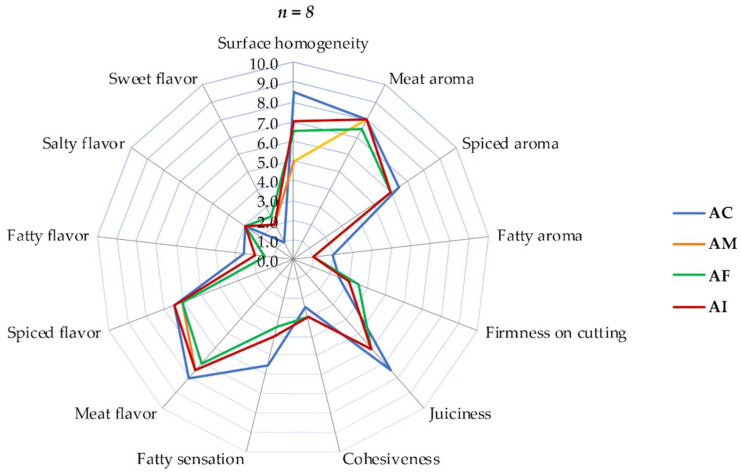
Sensorial profile of pork and chicken meatballs performed by semi-trained panelists (*n* = 8). Control AC meatballs (full fat, no fiber added), meatballs with inulin AI (reduced fat and added inulin dissolved in water), meatballs with fructo- oligosaccharides AF (reduced fat and with added FOS dissolved in water), and meatballs with the AM mixture (reduced fat and added inulin mixture and FOS dissolved in water).

Figure 4 shows the sensorial profile of pork and chicken meatballs accomplished by eight semi-trained panelists. The AC meatballs had the greatest homogeneity, fatty aroma, fatty sensation, juiciness, and meat flavor. The AI, AF, and AM meatballs had greater sweet flavor intensities than AC, as sweetness is characteristic of inulin and oligo-fructose. The challenge in the development of reduced-fat meat products is to obtain the texture and flavor which is as close as possible to those foods with high fat content. Despite the differences between meatballs with fiber and control meatballs, AI presented the sensorial characteristics most closely related to those of AC; for this reason, it was selected for the performance of hedonic sensorial tests and the determination of final consumer acceptance.

### 3.3. Selected Meatball (AI) Sensorial Evaluation with Consumers (Home-Based Test)

**Figure 5:**
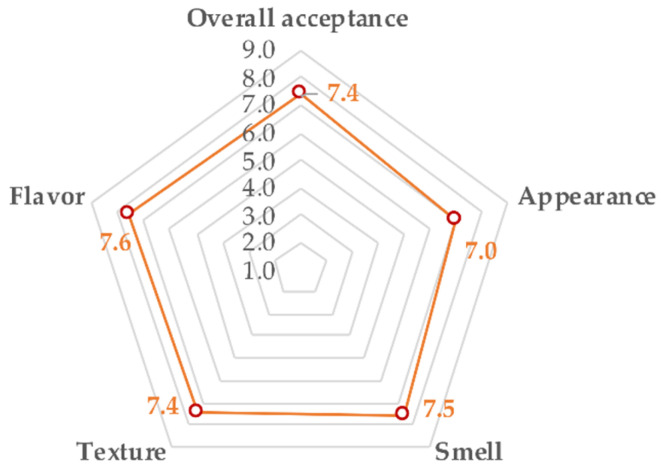
Radial graph of the sensorial characteristics of reduced-fat, preservative-free (AI) pork and chicken meatballs performed by consumers (*n* = 84) via a hedonic test.

The objective of the present investigation with consumers was to establish the acceptability of reduced-fat pork and chicken meatballs with added inulin. On a nine-point hedonic scale (1 = I strongly dislike them, 9 = I like them very much), consumers indicated an overall product acceptance of 7.4, appearance of 7.0, smell of 7.5, texture of 7.4, and flavor of 7.6 (Figure 5), indicating high consumer product acceptance.

**Figure 6:**
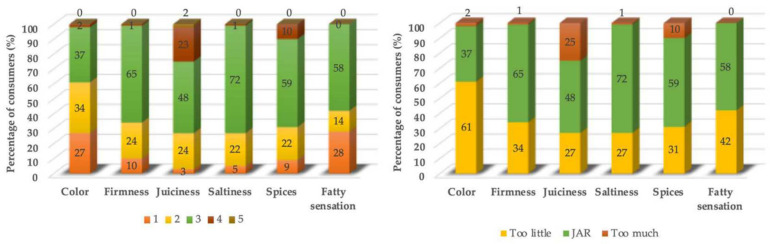
Results of the Just About Right (JAR) scale for reduced fat, preservative-free meatballs (AI) performed by consumers (*n* = 84).

The evaluated JAR scale identified that the toughness and salt levels were correct, provided that the spice and fat content were also at the right levels for the majority of the population. Exactly 48% of the consumers evaluated indicated that the juiciness was adequate, while 26% considered it to be very high, and 27% very low. Further, 61% of consumers considered the meatball to have a very light color, while 37% considered it adequate (Figure 6). This parameter is very dependent on the characteristics of raw materials and meatball color associations with beef, which is darker. Despite the addition of natural coloring to the formulation, in order to improve the color intensity, without being too near to the color of beef meatballs. This study determined that a more intense color is preferred.

The penalty analysis was performed to determine the influence of color, toughness, juiciness, saltiness, spice intensity, and fatty flavor on the overall acceptance of the meatballs, in order to guide the development of pork and chicken meatballs. Table 8 shows the penalty analysis. Observe that there was statistical significance (*p* < 0.05) in the penalty for the very light color, low spice levels, and low fatty flavor, which generated an impact on consumer acceptance. If one or several of these parameters were improved, the overall product acceptance (7.4) could increase. Specifically, it is recommended that the product color be improved, with an increased proportion of the natural color used.

### 3.4. Nutritional Composition of Selected Meatballs (AI)

The nutritional composition of pork and chicken meatballs, with the inclusion of inulin, is shown in Table 9. The initial formulation was created, considering Technical Colombian Regulation NTC 1325 [42], for premium products. In the table, the characteristics corresponding to protein above 14% and fat below 28% are corroborated. This characteristic is attributed to the fact that the pork loin and chicken breast employed in this formulation are lean meats with high protein content.

The combination of two protein types (pork loin and chicken breast) permitted the meatballs obtained in the present study to be considered an excellent source of protein, with 17.94 g of protein in 100 g of product. Authors such as Aripin et al. [75] reported the preparation of meatballs with chicken and duck meat in various concentrations, to obtain low protein content (from 5.88–10.45%). Yeung and Huang [76] prepared pork meatballs with the incorporation of different additives, to obtain elevated protein levels (from 15.74–16.04%).

The total and saturated fat in meatballs with inulin represent just 10% of daily intake (based on a 2000 kcal diet), as compared to commercial meatballs, which provide over 17% of the daily intake value and saturated fat total. The sodium content of the prepared meatballs was low, as per 100 g of product, 340.75 mg is ingested, representing 17.03% of the intake established by the World Health Organization, which is 2000 mg of sodium per day, per person. In commercial meatballs, however, one ingests 500 mg of sodium per 68 g of product, or 25% of the recommended intake.

The result of fiber content in the inulin meatballs was unexpected, as 3.5% of inulin was added during preparation, and only 0.56 g of inulin is reported in 100 g of product. There may be inulin losses throughout the cooking process which oscillate within 3–5%, and this yields content of greater than 3 g of inulin in 100 g of product. Angiolillo et al. [26] reported similar results on the inclusion of inulin, FOS, and oat bran to hamburger meat, where the fiber analysis came out null for meat with FOS and inulin, and at 0.21 g per 100 g of meat with the inulin and oats.

## 4. Discussion

The consumer sensorial perception survey for meatball consumption permitted confirmation that claims are an important part of the development and labelling of a food. Meatballs with additional claims, such as reduced-fat, added fiber, or preservative free have better consumer perceptions than those without claims on their label. In the case of preservatives, it was shown that, despite the fact that claims of reduced-fat and extra fiber are considered the same for healthy components, this is not part of the consumption perception, as there is a higher probability that an individual consumes meatballs labeled preservative free rather than added fiber.

In the preparation of the four meatball formulations (AC, AI, AF, and AM), significant differences were found in terms of certain physicochemical and sensorial parameters measured. AC was the sample with the lowest humidity, because in the reduced fat formulation, the replacement of fat was accompanied by the addition of fiber dissolved in water. As such, the amount of water present in AC was lower than in the other samples. Despite the differences in the amount of added water, the available water is equal in all formulations, as fiber has a high-water retention capacity, generating an interaction with the water added to these formulations. In the present study, neither water activity nor pH changed owing to fat reduction, similar to that which occurred with the addition of short-chain fructo-oligosaccharides in low-fat sausage [77].

The samples’ losses of weight during cooking in AI, AF, and AM indicate that, despite the fibers’ water retention capacity [78], there is a portion of water that was not immobilized by the interactions created in the gel network, permitting their liberation to the external cooking media. Some researchers have reported this characteristic as a technological disadvantage of inulin [30], and others have found that inulin improves cooking performance and presents advantages in emulsion stabilization [28,79,80]. In certain cases, it has been observed that soluble, insoluble, and starch fiber mixtures result in a reduction in the weight loss of hamburgers and meat emulsions [26,81].

In terms of color, the luminosity of the internal part of meatballs AI, AF, and AM was greater than in AC. This is likely due to the brightness provided by the fiber gels in the meat matrix. Huang et al. [82] reported that there were no color changes in sausages with added fiber: inulin, wheat fiber, and oat fiber, while Menegas et al. [83] observed a tendency toward lighter and reddish coloring in fermented chicken sausages with added inulin and corn oil. However, Cáceres et al. [77] reported decreases in luminosity values in reduced-fat sausages with added fructo-oligosaccharides. Paglarini et al. [84] found that the addition of a high-fiber emulsion gel containing inulin in Bologna increased L* (lightness) values and reduced a* (redness/greenness) values comparing to control treatment.

The texture, measured as the firmness in meatballs (Kramer) was significantly higher in control meatballs than in those with added fiber. The firmness of cut and cohesiveness measurements of the panelists did not present significant differences between the AI, AF, AM, and AC treatments, while juiciness was highest in AC. Huang et al. [82] reported an increase in firmness in sausages with added wheat and oat fiber, while those with added inulin did not present texture differences. Keenan et al. [79] found that firmness values increased with the concentration of inulin, and panelists also rated products with inulin to be less tender. Other authors establish that the addition of FOS to meat products permitted slightly more tender products than those without FOS or fat reduction [77].

Inulin is used as a fat replacement, as in the presence of water, it develops particulate gels which improve the product texture, with a mouth feel similar to that produced by fat [85]. In the present study, it was observed that, depending on the added fiber and water content, as well as the fat replaced, lower fat content may reduce juiciness, causing greater toughness. However, fiber may mimic this characteristic, to a certain point, and provide tenderness. It may also generate a mass that is excessively tender, so as to produce unpleasant sensorial characteristics that may significantly affect product homogeneity. For this reason, control meatballs had a more defined form than those with added fiber.

The addition of fiber to meat matrices induces the formation of stable gel networks [25,85], which permits the improvements of meatball rheology and texture characteristics, in this case. However, many of these characteristics are dependent on the remaining food matrix components, emulsion formation, meat grind, product cooking, and especially the amount of water added in preparation. Considering the physicochemical characterization, results with trained panelists permitted the selection of the formulation with sensorial characteristics most like the control meatballs, which was that with inulin (AI), which was then put to consumer sensorial analysis.

The results of the reduced-fat pork and chicken meatball (AI) sensorial analysis performed by consumers showed high levels of product acceptability in terms of overall acceptance characteristics, appearance, smell, texture, and flavor. The flavor of spices perceived by consumers was found to be at the correct level.

For consumers, the salt level was also found to be at the correct level (JAR) Meat products are characterized by high levels of sodium chloride (table salt), as this may be used, industrially, at concentrations of 1–2.5% in cooked products, and from 2–6% in raw, cured products [86]. In the formulation, 1% salt (1 g/100 g of mixture prior to cooking), reflecting a sodium content in the final product that is not high. This responds to public health policies that have established regulations in different countries to monitor sodium consumption, thus creating a need in the food industry to reduce sodium in their products.

The fatty flavor was adequate (JAR) for consumers and reduced-fat meatball acceptance was good. Juiciness is another important characteristic in the development of reduced-fat foods. The results were inconclusive for consumer perceptions thereof, as 48% indicated that the product’s juiciness was adequate, and 27% reported it to be very low, and 26% reported it to be quite high. However, the population rated the texture parameter as 7.4 on a hedonic scale of 1–9, which indicates good acceptability.

The penalty analysis indicated that the light color, minimal spice flavor, and minimal fat flavor characteristics are penalized highly by the consumer, when providing overall product acceptance ratings. Less than 20% of consumers identified a “minimal spice flavor” or “minimal fat flavor”, for which reason improvements to these parameters are not proposed. Most consumers indicated that the meatballs presented a “very light color”, which statistically penalized the overall acceptance of the product. As such, a formulation with greater natural color content could be proposed.

This product is reduced in fat, and an excellent source of protein. Saturated fat and sodium were both below those limits established for food labelling regulations. It was expected that 90% of the added fiber would be retained, since theoretically only a maximum of 5% of soluble fiber is lost in cooking processes. Although the fiber generated a positive technological effect, the fiber content at the end of cooking was very low. It would be important to set the modifications to the fiber quantification methodologies after meat cooking processes.

The COVID-19 pandemic situation generated restrictions for access to laboratories and forced us to change the methodology of sensory analysis with consumers from a room location test to a home test. Initially, it was planned to evaluate the 4 formulations of meatballs with consumers, but finally, the evaluation was done with trained panelists. Only one formulation was selected to evaluation with consumers.

In future research it would be important to determine the effect of inulin in other types of products such as hamburgers, sausages and nuggets made from pork and chicken. This would allow identifying if it is possible that inulin, in addition to the technological effect on fat reduction, also allows obtaining a food that is a good source of fiber.

The influence of an ingredient in the development of reduced-fat foods should be accompanied by a shelf-life study of this food, especially when there are no preservatives in its formulation. It is recommended to always perform stability studies to complement the effect of these ingredients. In this case, shelf-life studies with the selected formulation were carried out and will be published later.

## 5. Conclusions

The declarations preservative free, reduced fat, and added fiber are of great importance for consumers when they consider meatballs as health food. However, there is greater probability of meatball consumption when they claim to be “preservative-free”, as compared to others that claim to be “reduced-fat” or a “good source of fiber”. Definitively, a meatball with no nutritional claim presents minimal perceptions of a healthy food, and has reduced probability of consumption.

The acceptance of the appearance of pork and chicken meatballs is predetermined by the association with the color of beef meatballs. Even though this does not represent a significant parameter on evaluation of their general sensorial acceptance and the purchase decision, this may increase appearance acceptance, if higher concentrations of natural color are used in the formulation, so as to obtain a food product with a darker brown color.

The inclusion of inulin as a fat substitute in the preparation of pork and chicken meatballs (3.5 g of fiber/100 g of mixture) permits the imitation of the technological properties of fat, without significantly affecting the sensorial characteristics of the food.

## Figures and Tables

**Table 1:** Scales used for the evaluation meatball consumption perceptions.

Perception of Healthiness		Probability of Consumption		Product Opinion	
Unhealthy	Extremely healthy	Very unlikely to try	Very likely to try	I don’t like them at all	I like them very much
1	7	1	7	1	7

**Table 2:** Formulation of pork and chicken meatballs with added inulin and/or fructo-oligosaccharides.

Ingredients	Amount (g/100 g of Raw Meatballs)			
	AC	AF	AI	AM
Chicken breast	30.0	30.0	30.0	30.0
Lean pork loin	39.7	39.7	39.7	39.7
Pork fat	16.5	6.0	6.0	6.0
Corn flour	3.5	3.5	3.5	3.5
Water	6.0	6.0	6.0	6.0
Fiber water	0.0	7.0	7.0	7.0
FOS	0.0	3.5	0.0	1.8
Inulin	0.0	0.0	3.5	1.8
Salt	1.0	1.0	1.0	1.0
Ground garlic	0.7	0.7	0.7	0.7
Powdered onion	0.5	0.5	0.5	0.5
Ground oregano	0.2	0.2	0.2	0.2
Ground thyme	0.1	0.1	0.1	0.1
Ground bay leaf	0.1	0.1	0.1	0.1
Ground basil	0.1	0.1	0.1	0.1
Ginger	0.1	0.1	0.1	0.1
Ground black pepper	0.1	0.1	0.1	0.1
Carl natural color	1.5	1.5	1.5	1.5

**Table 3:** Definitions and references of descriptive terms for meatballs, which correspond to the ends of the measurement scales.

Attribute	Definition	Reference
Surface homogeneity	Visual perception of homogeneity on the surface of the meatball.	Slight: Cracked meatball. Meatball formulation without corn flour High: Well-formed meatball. Meatball formulation with corn flour (8.5%)
Meat aroma	Intensity of the aroma characteristic of chicken and pork.	None: Distilled water Strong: Chicken breast and/or pork loin cooked in water
Spiced aroma	Intensity of the aroma characteristic of the spices (oregano, thyme, bay leaf, basil, pepper, garlic, onion).	None: Spiceless meatball Strong: Meatball with 3.5% spices
Fatty aroma	Intensity of the aroma of fat.	None: Chicken breast cooked in water Strong: Fried bacon
Meat flavor	Intensity of the flavors characteristic of chicken and pork.	None: Distilled water Strong: Chicken breast and/or pork loin boiled in water
Spiced flavor	Intensity of the flavors characteristic of the spices (oregano, thyme, bay leaf, basil, garlic, onion).	None: Spiceless meatball Strong: Meatball with 3.5% spices
Fatty flavor	Intensity of the flavor of fat.	None: Chicken breast boiled in water Strong: Fried bacon
Salty flavor	Intensity of the salty flavor, associated with the presence of salt.	None: Saltless meatball Strong: Meatball with 2.5% salt
Sweet flavor	Intensity of the sweet flavor.	None: Sugarless meatball Strong: Meatball with 14% sugar
Firmness on cutting	Degree of meatball firmness when cut. This is closely related to cohesiveness. It is rated firm and cohesive when the meatball is cut with a knife without any loss of structure or crumbling.	Slight: Commercial meatball High: Meatball formulation with corn flour (8.5%)
Juiciness	Perception of water absorbed or freed from the meatball during chewing. A succulent meatball frees a great deal of liquid as the product is chewed, and one that is not frees very little liquid, producing the sensation of a dry product.	Slight: Meatball formulation with corn flour (8.5%) High: Commercial meatball
Cohesiveness	Degree to which the meatball stays together or compact.	Slight: Commercial meatball High: Meatball formulation with corn flour (8.5%)
Fatty sensation	Amount of fat perceived in the mouth, especially on the palate and lips.	None: Chicken breast boiled in water High: Fried bacon

**Table 4:** Paired comparison test for meatballs perception of healthiness.

Sample 1–Sample 2	Test Statistic	Std. Error	Std. Test Statistic	Sig.	Adj. Sig.
Traditional-Added fiber	314.540	28.965	10.859	0.000	0.000
Traditional-Reduce fat	338.440	28.965	11.684	0.000	0.000
Traditional-Preservative free	366.763	28.955	12.662	0.000	0.000
Added fiber-Reduce fat	23.900	28.955	0.825	0.409	1.000
Added fiber-Preservative-free	−52.222	28.955	−1.803	0.071	0.428
Reduce fat-Preservative free	−28.322	28.955	−0.978	0.328	1.000

**Table 5:** Paired comparison test of meatball probability of consumption.

Sample 1–Sample 2	Test Statistic	Std. Error	Std. Test Statistic	Sig.	Adj. Sig.
Traditional-Added fiber	120.381	28.781	4.183	0.000	0.000
Traditional-Reduce fat	166.140	28.738	5.781	0.000	0.000
Traditional-Preservative free	221.446	28.803	7.688	0.000	0.000
Added fiber-Reduce fat	45.759	28.781	1.590	0.112	0.671
Added fiber-Preservative-free	−101.065	28.846	−3.504	0.000	0.003
Reduce fat-Preservative free	−55.306	28.803	−1.920	0.055	0.329

**Table 6:** Physicochemical pork and chicken meatball parameters.

Physicochemical Parameter	Treatment			
	AC	AI	AF	AM
Humidity (%wb)	59.9 ± 0.000 ^a^	65.9 ± 0.000 ^b^	65.6 ± 0.002 ^b^	65.8 ± 0.001 ^b^
pH	6.008 ± 0.010 ^a^	6.032 ± 0.004 ^a^	6.028 ± 0.002 ^a^	6.016 ± 0.007 ^a^
Water activity	0.987 ± 0.000 ^a^	0.986 ± 0.001 ^a^	0.989 ± 0.001 ^a^	0.988 ± 0.001 ^a^
Kramer maximum force (N)	248.275 ± 8.049 ^a^	220.689 ± 6.956 ^b^	217.305 ± 4.651 ^b^	219.618 ± 5.414 ^b^
Weight loss (%)	11.96 ± 0.44 ^a^	17.16 ± 4.99 ^b^	17.59 ± 2.87 ^b^	19.50 ± 0.98 ^c^
Color				
Crust				
L*	59.420 ± 0.183 ^a^	60.171 ± 0.037 ^a^	60.081 ± 0.037 ^a^	59.523 ± 0.085 ^a^
a*	6.342 ± 0.009 ^a^	6.522 ± 0.0497 ^a^	6.411 ± 0.012 ^a^	6.513 ± 0.011 ^a^
b*	13.136 ± 0.007 ^a^	12.793 ± 0.026 ^a^	12.428 ± 0.137 ^a^	12.723 ± 0.174 ^a^
C*	14.591 ± 0.012 ^a^	14.369 ± 0.016 ^a^	13.985 ± 0.129 ^a^	14.297 ± 0.163 ^a^
h*	64.167 ± 0.001 ^a^	62.950 ± 0.281 ^a^	62.699 ± 0.163 ^a^	62.893 ± 0.204 ^a^
Center				
L*	58.471 ± 0.014 ^a^	59.455 ± 0.172 ^b^	59.222 ± 0.007 ^b^	59.194 ± 0.021 ^b^
a*	6.620 ± 0.007 ^a^	6.821 ± 0.057 ^a^	6.826 ± 0.064 ^a^	6.818 ± 0.016 ^a^
b*	14.280 ± 0.158 ^a^	14.435 ± 0.040 ^a^	14.489 ± 0.064 ^a^	14.124 ± 0.131 ^a^
C*	15.741 ± 0.166 ^a^	15.969 ± 0.003 ^a^	16.020 ± 0.093 ^a^	15.685 ± 0.126 ^a^
h*	65.116 ± 0.0253 ^a^	64.709 ± 0.054 ^a^	64.770 ± 0.034	64.235 ± 0.046 ^a^
Proximate composition				
Fat (g/100 g)	15.715 ± 0.488 ^a^	6.65 ± 0.424 ^b^	6.145 ± 0.615 ^b^	7.095 ± 0.898 ^b^
Protein (g/100 g)	18.205± 2.751 ^a^	17.940 ± 0.424 ^a^	18.815 ± 0.615 ^a^	18.320 ± 0.898 ^a^
Ash (g/100 g)	1.455 ± 0.035 ^a^	1.450 ± 0.042 ^a^	1.45 ± 0.000 ^a^	1.51 ± 0.014 ^a^

Data averages shown ± standard deviation. Different superscript letters in the same row indicate different significance (*p* < 0.05). Lightness (L*), redness+/greenness (a*), yellowness+/blueness (b*), Chroma (C*) and Hue angle (h*).

**Table 7:** Microbiological meatball analysis.

Microbiological Analysis	NTC 1325	Result
Mesophilic aerobes, CFU/g	<100,000	86.67
Coliforms, CFU/g	100–500	13
*S. aureus* positive coagulase, CFU/g	<100	<100
*Salmonella* detection, 25 g	None	None
*C. perfringens* reducing sulphite spores, CFU/g	<10–100	<10
*Listeria monocytogenes*, 25 g	None	None
*E. coli*, g	<10	None

**Table 8:** Penalty analysis for reduced-fat pork and chicken meatballs without preservatives (AI).

Variable	Level	%	Mean Drops	*p*-Value for the Endpoint	Penalties	*p*-Value for the Attribute
	Very clear	60.76	0.654	0.012		
Color	JAR	36.71			0.619	0.017
	Very dark	2.53	−0.241			
	Very soft	34.18	0.399	0.139		
Firmness	JAR	64.56			0.403	0.128
	Very firm	1.27	0.510			
	Not juicy	26.58	1.197			
Juiciness	JAR	48.10			0.865	0.000
	Very juicy	25.32	0.516			
	Saltless	26.58	0.947			
Saltiness	JAR	72.15			0.887	0.001
	Very salty	1.27	−0.386			
	Very little	30.38	0.784	0.006		
Spiced flavor	JAR	59.49			0.617	0.016
	Very intense	10.13	0.117			
	Very low	41.77	0.630	0.013		
Fat sensation	JAR	58.23				
	Very high	0.00				

**Table 9:** The nutritional composition of reduced fat preservative-free pork and chicken meatballs (AI).

Nutrient	Amount
Protein (g/100 g)	17.94 ± 0.0
Fat (g/100 g)	6.65 ± 0.30
Saturated fat (g/100 g)	2.325 ± 0.18
Monounsaturated (g/100 g)	2.865 ± 0.03
Polyunsaturated (g/100 g)	1.46 ± 0.10
Trans isomers (g/100 g)	0.035 ± 0.02
Ash (g/100 g)	1.44 ± 0.01
Iron (mg/100 g)	1.615 ± 0.11
Cholesterol (mg/100 g)	5.253 ± 3.50
Sodium (mg/100 g)	340.75 ± 34.21
Fiber (g/100 g)	0.56 ± 0.01

## Data Availability

Data is contained within the article.
